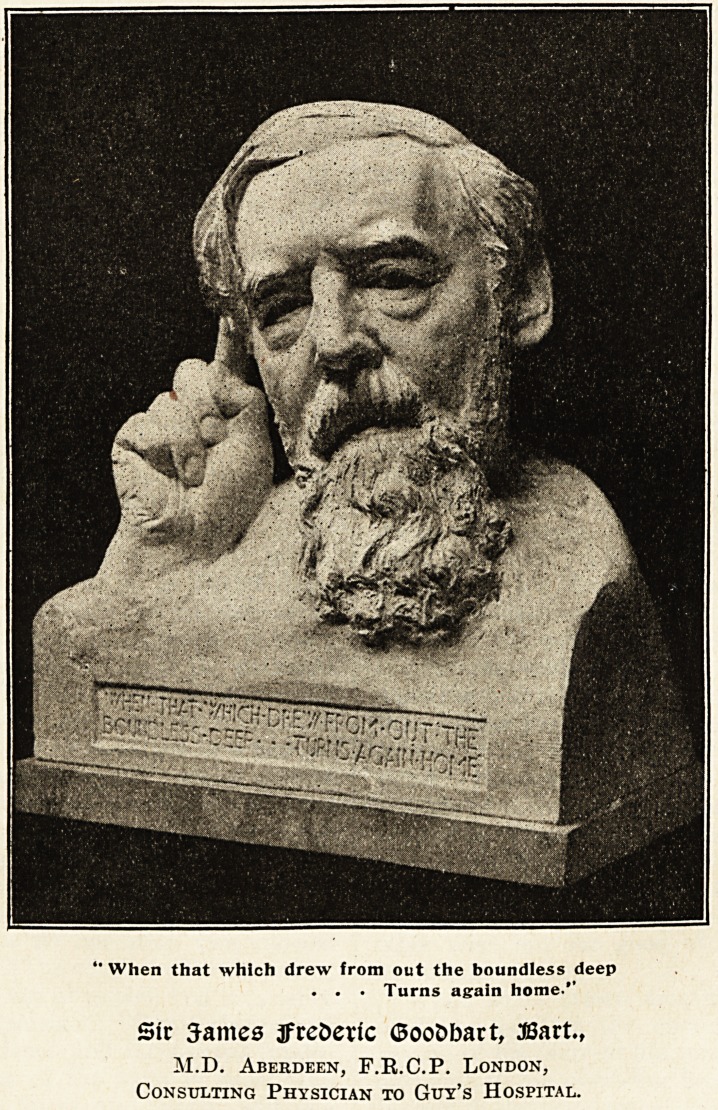# Sir James Frederic Goodhart: A Great Physician and Gentleman

**Published:** 1916-06-03

**Authors:** 


### A GREAT PHYSICIAN AND GENTLEMAN.

### SIR JAMES FREDERIC GOODHART, Bart., M.D. Aberdeen, F.R.C.P. Lond.

THE suddenness to the majority of his friends and the public of the death of Sir James Goodhart is characteristic of his whole career. He was never in the limelight, which he avoided as he avoided poison. His whole energies, interests, time, and thoughts were devoted to his profession and his patients. In times of struggle, of prosperity, and of continued success, Sir James remained the quiet, thorough, capable, untiring, devoted student who pursued the even tenor of his way, quite unmoved, and wholly irrespective of the circumstances of the moment. Last fall he had a chill which laid him up, and the effects of which hung about him to his discomfort until his death. Yet he never complained even to his intimate friends, but continued his work, was always cheerful, continuously interested in his patients, their affairs and well-being, and seemed through it all, to them at any rate, to be in reasonably good health and spirits. Thoroughness, self-suppression, quiet efficiency, unselfish interest in and untiring devotion to the interests of others, were marked features of his conduct, his character, and his methods. During the last few weeks of his life he was an invalid, and his symptoms increased in seriousness, but no inkling of this was allowed to reach the public. Only one short intimation a few days before his death appeared in the Press. We have known in the past many great physicians and members of the profession; we do not remember one whose death occurred after serious illness without daily bulletins finding their way into the papers. And we welcome this exception because it is so characteristic of the retiring modesty of a delightful character which has made its mark on the world and the profession of medicine. It is interesting, too, that this great diagnostician, probably one of the greatest of British physicians, should have passed away, as we understand, without declared diagnosis, until the *post-mortem*.

"When that which drew from out the boundless deep . . . Turns again home."

**Sir James Frederic Goodhart, Bart.,**
M.D. ABERDEEN, F.R.C.P. LONDON,
CONSULTING PHYSICIAN TO GUY'S HOSPITAL.

It was in 1871 that James Frederic Goodhart took his M.B., C.M. degree at Aberdeen with the highest honours, and special honours for his graduation thesis. On his way down from the North he stayed as a candidate for the house - physiciancy to the Queen's Hospital, at Birmingham, and was elected. He made a very favourable and deep impression upon some of those who met him on that occasion, and after the election he was asked whether he had fully considered the possibility of an opening for himself in London, where his undoubted abilities would ensure him the widest opportunity for a successful career. He agreed to consider fully this aspect of the future, from his own point of view, on his return to London, and the appointment at Birmingham was kept open for him for a short time to enable this to be done. In the result he determined to remain in London, where he speedily became recognised, and his success was continuous and uninterrupted. From boyhood upwards his was a shy and retiring nature. It was his modesty, no doubt, which caused him to apply for the post of house physician at Birmingham.

James Frederic Goodhart was born on October 24, 1845, in Camden Road, where his father was then in practice as a doctor. When six years old he had the misfortune to lose his father, and his mother was left with four sons and a daughter, James Frederic being the second son. It is very interesting to record the fact, which we have no doubt the authorities of Epsom College will duly note and treasure as of historical interest, that Goodhart, at the age of ten, went there at its opening as one of the first boys. School life could never be really happy to one of his shy and retiring nature, but he did exceedingly well at Epsom and won several prizes. He entered as a student at Guy's Hospital, where he pursued his career for some time, thence going to Aberdeen, where he took his degree, then returning to London. At Guy's he held successively most of the junior appointments, became lecturer in pathology, then assistant, and afterwards full physician. In 1899 it became clear to him that the great pressure of his private work made it essential, to his infinite regret, to resign the physicianship at Guy's as an act of simple justice to his beloved hospital, its students, and the claims of younger men, who could devote the necessary time and do full justice to the patients and the teaching at that great centre of medicine.

He was ever most thorough and painstaking in his work, and his devotion in earlier years to pathology laid the foundation for the great eminence he attained as a master of diagnosis, whose treatment of disease was equalled by few and surpassed by none of his contemporaries. In addition to his work at Guy's, he became a member of the honorary staff of the Evelina Hospital, where he rapidly attained great success as a specialist in children's diseases. In this field of work he was in his true element, for he loved children and he loved his work. His book on "The Diseases of Children," of which the tenth edition was published in 1913 (with Dr. G. F. Still), and his very valuable articles in Sir Clifford Allbutt's "System of Medicine" have world-wide reputation. Among a number of treatises the best known is probably "Common Neuroses," the Harveian Lecture in 1891 with a second edition in 1894. The great success and high reputation which the book on children's diseases brought him is said occasionally to have led laymen to regard him as a children's specialist and to hold lightly his clinical wisdom in general medicine. But no one in the profession ever fell into this grave error. To all practising medical men he was one of the wisest counsellors and most trusted consultants in cases of doubt and difficulty. He was a most attractive lecturer and speaker, and members of the profession used to flock to hear him on the far too few occasions on which he spoke. His style had a convincing frankness about it. He possessed an admirable spirit of humour, and was altogether so kindly and good a man, and such a gentleman, that he won the affection of everybody who had the privilege of knowing him, and especially of those who were his friends and intimates.

It is very interesting to look back over the forty-five years during which Sir James Goodhart took an active and increasing part in professional work. The changes which have come over the profession during that period have been many and remarkable. Not the least of them has been the enormous development in surgery and its strength and increasing volume, which, it is to be hoped, has at length attained its zenith. There can soon be too much surgery in an age of increasing hurry and impatience when people of all classes have come to regard the surgeon's knife as the shortest cut to the speediest removal of most bodily ailments and troubles. The passing of the National Insurance Acts, and the institution of the panel system, have proved anything but an advantage to the masses of the people in matters of treatment. When Sir James Goodhart commenced his career the great stand-by and safeguard to all classes of the community when ill was the predominant position of, the authority exercised by, and the deserved confidence extended to the general practitioner of medicine. He was not only the family doctor, but the family friend, who was deservedly trusted and almost invariably consulted in all times of anxiety and difficulty. The modern innovations just referred to threaten the existence not only of the general practitioner, but that of the consulting physician too. Such threatenings are undeniably dangerous, and their eventuation in the termination of the existence of either or both could not fail to be wholly evil in their effects upon the welfare, the general health, and, in many cases, we make bold to say, the long life of an increasing number of people of all classes.

Accurate diagnosis should ever be the test of full knowledge, as it should constitute the basis of confidence for the patient towards the practitioner. The steady extension of a policy which regards diagnosis in the old sense as out of date, because the exploratory incision can be claimed in an ever-increasing number of cases as the shortest method of revealing the true causes of the patient's troubles, points to results, as experience has already demonstrated, that can be and ought to be avoided in the treatment of disease by members of a learned profession. The true safeguard and remedy against excessive indulgence in the surgical policy referred to are the steady maintenance and upholding of the physician and the general practitioner, whose presence is essential to the welfare and to the defence of the rights of the patient. In the old days of barber-surgeons no operation was allowed to be undertaken without the consent of a physician, and if it was agreed to, the physician had invariably to be present at the operation. Our forbears and sires were men of prudence and far-sighted intelligence. They were not of the type of men who sacrifice everything to hurry and speed, and one of the lessons of the life of a great physician like Sir James Goodhart, who attained such splendid results as a practitioner from his great diagnostic gifts and perfected practical knowledge of disease, is plain. It is undoubtedly for the profession and the public to remember that there are two great branches of the profession, those of medicine and surgery; that they are interdependent and self-supporting; and that the abrogation or even the undue prominence of either by or over the other must ultimately end in disaster not only to both, but to the profession and also to the people of all classes.

Of academical distinctions, as well as of the esteem and admiration of his professional brethren, Sir James Goodhart had enough and to spare. From the time of his first graduation he never looked backward: perhaps the most noteworthy later landmark of this side of his career was the honorary degree of LL.D. conferred by Aberdeen University in 1899. He was at the time of his death one of the select company of medical baronets (cr. 1911), of whom there are at present eighteen. Sir James Goodhart never took an active part in professional politics or in public affairs, to which some leaders of the profession frequently devote much of their time. For nineteen years he was consulting physician to Guy's Hospital; he gave his services gladly to the Royal Hospital for Incurables, to the Female Orphan Asylum, and was a member of the consulting staff of King Edward VII. Sanatorium. He served for a time the office of examiner in medicine at the Royal College of Physicians, London, and the Royal College of Surgeons, England, but when a general wish was expressed that he should be a candidate for the presidency of the Royal College of Physicians he declined the honour, his reasons being that there were others with higher claims to whom the office would be of infinite value, and whose services to medicine, in his opinion, deserved greater recognition than they had then received. All through his professional career, and indeed throughout his whole life, the underlying principle which constituted, we have no doubt, the secret of his immense success, was love. Love ruled his life. It was the genesis of his first successes as a specialist in children's diseases, for he was devoted to children. He loved his work; he loved his family; he loved his friends, with a peculiar tenderness which was winsome and inspiring. It may be said with truth that James Frederic Goodhart never in the whole course of his life gave avoidable pain to a living thing. He never did a shabby action, nor was he capable of taking the smallest advantage of another or of ever assuming any attitude towards his fellows except one of earnest desire for their welfare and the devotion of his whole energies to help them to promote it. It is the simple truth that he had no other wish but the good of those with whom he came into contact.

When some fifteen months ago Lady Goodhart died and he lost the cherished friend and companion of his married life, one of the most touching elements in his character became noticeable by his friends in the deep-seated continuance of his loving affection for her who had been his life companion. The shadow of her dimmed his energies and diminished his interests, facts which, coupled with the shock and horror that he felt at the war and all the cruelties and troubles it has brought in its train, no doubt contributed to the premature termination of a great life. That life was nobly and unselfishly spent in promoting the health, the happiness, and the welfare of those with whom he came into contact. Goodhart was a great and good man.

## Figures and Tables

**Figure f1:**